# Determination of Polyhexamethylene Biguanide Hydrochloride Using a Lactone-Rhodamine B-Based Fluorescence Optode

**DOI:** 10.3390/molecules25020262

**Published:** 2020-01-09

**Authors:** Akane Funaki, Yuta Horikoshi, Teruyuki Kobayashi, Takashi Masadome

**Affiliations:** Department of Applied Chemistry, Faculty of Engineering, Shibaura Institute of Technology, Toyosu, Koto-ku, Tokyo 135-8548, Japan

**Keywords:** optode, polyhexamethylene biguanide hydrochloride, lactone-rhodamine B, contact-lens detergent

## Abstract

A new determination method for polyhexamethylene biguanide hydrochloride (PHMB) using a lactone-rhodamine B (L-RB) based fluorescence optode has been developed. The optode membrane consists of 2-nitrophenyl octyl ether as a plasticizer, L-RB, and poly (vinyl chloride). The optode responds to tetrakis (4-fluorophenyl) borate, sodium salt (NaTPBF) in the μM range. The fluorescence intensity of the L-RB film for PHMB solution containing 20 μM NaTPBF decreased linearly as the concentration of the PHMB solution increased in the concentration range from 0 to 8.0 μM, which shows that PHMB with a concentration range of 0 to 8.0 μM is determined by the L-RB film optode. The concentration of PHMB in the contact lens detergents by the proposed method was in accord with its nominal concentration.

## 1. Introduction

Polyhexamethylene biguanide hydrochloride (PHMB), a kind of a cationic polyelectrolyte (CP), is very useful for disinfectants in a contact lens detergent (CLD) and sanitizers for swimming pools. Many analytical methods for PHMB have been studied [1,2,3,4,5,6,7,8,9,10,11,12]. However, the lower determination concentration of several analytical methods for PHMB is above a few ppm, and the methods have very tedious operation procedures and use very toxic reagents frequently. Furthermore, the methods were not applied to the measurement of PHMB in commercially available CLDs because the concentration of PHMB in the commercially available CLDs is ca. 1.1 ppm (5.0 μM). A highly sensitive HPLC method with a solid phase extraction and an evaporative light scattering detector, whose lower detection limit is 0.1 ppm (0.45 μM), has been reported for the determination of PHMB in CLDs [4]. However, the apparatus of the method is very expensive and exaggerated. On the other hand, we have reported a new spectrophotometric flow injection analysis (FIA) method for the determination of PHMB and determined PHMB in the CLDs by the FIA method [11]. However, a large amount of reagent solution was consumed. Recently, Uematsu et al. reported a new promising determination method of PHMB using a glucose oxidase enzymatic reaction. The method is relatively simple and has high sensitivity (determination range of 0.05 to 0.4 ppm). However, the method utilizes a relatively high-cost enzyme such as a glucose oxidase and catalase [12].

Therefore, a simpler and low-cost analytical method with a low amount of used reagent solution and waste for the measurement of PHMB in CLDs using a chemical sensor is very helpful. The chemical sensor is very useful analytical tool, since it has the advantages of simple operation and relatively low cost. A highly sensitive electroanalytical sensor for PHMB by using adsorptive voltammetry as reported by Hattori et al. did not apply to the determination of PHMB in CLDs [6]. We have already reported an FIA method for a CP and an anionic polyelectrolyte (AP), utilizing the formation of an ion associate between a cationic polyelectrolyte and AP or an anionic surfactant (AS), and an ion-selective electrode (ISE) detector [13,14].

An analytical method of PHMB using an optode as a chemical sensor is very promising, because the optodes have several excellent advantages that are distinct from the electrochemical measurements such as the adsorptive voltammetric electroanalytical sensor and the ISE, in that the optodes are less susceptible to electrical noise, do not require a lead wire from their sensing membrane, and do not require a reference electrode for measurement. However, no research papers regarding the determination method for PHMB using an optode have been published so far.

Recently, we have reported an AS-sensitive optode based on a lactone rhodamine B (L-RB) film [15,16,17]. From the results, PHMB is expected to be determined with an optode responding to a hydrophobic anion such as AS, because PHMB will form an ion associate with the hydrophobic anion, and the concentration of the free hydrophobic anion is detected by the AS optode.

In this work, we have developed the determination method of PHMB in CLDs using an optode based on L-RB film.

## 2. Results and Discussion

### 2.1. Absorption Spectra, Excitation, and Fluorescence Spectra of an L-RB Film after Immersing to a NaTPBF Solution

The sample solution contains PHMB and a hydrophobic anion, NaTPBF (constant concentration). An increase of concentration of PHMB decreases the concentration of free NaTPBF, because PHMB forms an ion associate with NaTPBF quantitatively. PHMB is determined by measuring the decrease of concentration of free NaTPBF by the optode based on L-RB film.

**Figure 1:**
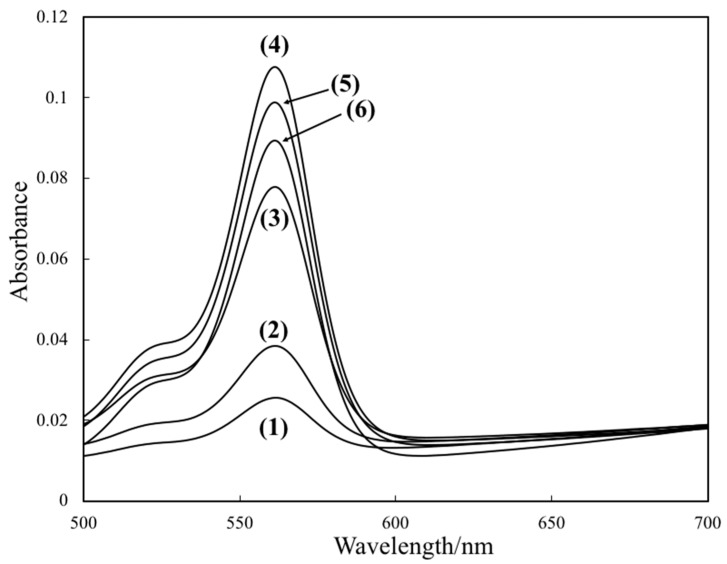
The absorption spectra of the lactone-rhodamine B (L-RB) film after it was immersed to a NaTPBF solution (pH 4.0, adjusted with a 0.1 M CH_3_COOH/CH_3_COONa buffer solution) for 30 min, respectively. Concentration of NaTPBF: (1) 0 µM, (2) 1.0 µM, (3) 5.0 µM, (4) 10.0 µM, (5) 50.0 µM, and (6) 100.0 µM.

**Figure 2:**
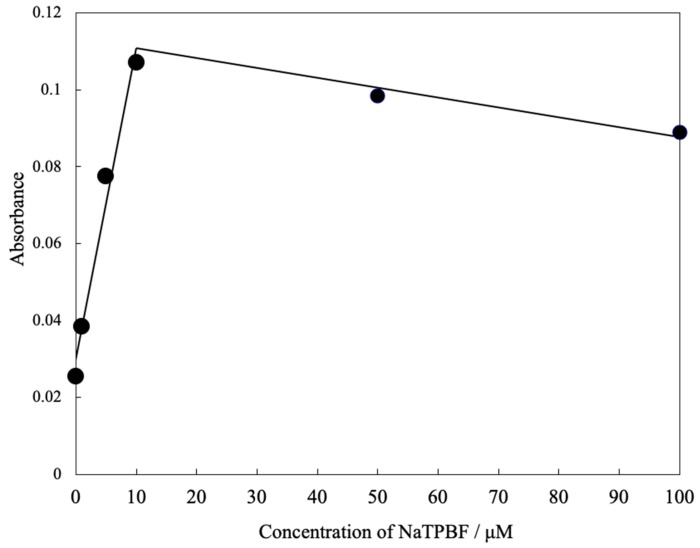
Calibration curve for NaTPBF obtained by using an L-RB-based optode.

**Figure 3:**
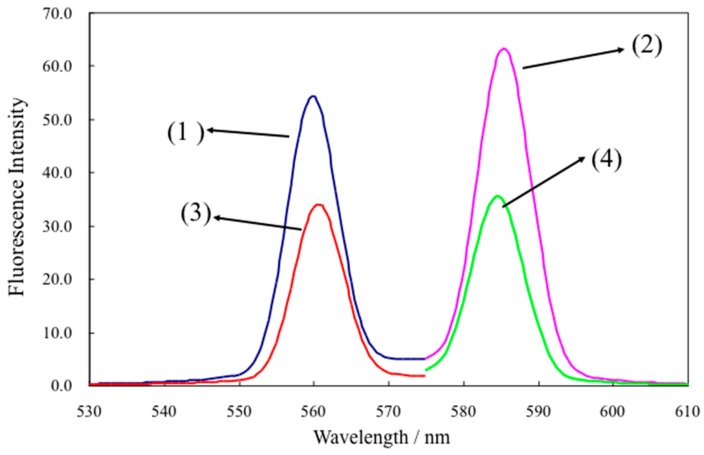
The excitation and fluorescence spectra of the L-RB film after it was immersing to a 5.0 µM NaTPBF solution (pH 4.0, adjusted with a 0.1 M CH_3_COOH/CH_3_COONa buffer solution) (1: excitation spectrum), (2: fluorescence spectrum), and that of the pH 4.0 buffer solution (0 µM NaDBS solution) (3: excitation spectrum), (4: fluorescence spectrum) for 30 min, respectively.

At first, we examined the response of the optode based on L-RB film to NaTPBF. Figure 1 illustrates the absorption spectra of the L-RB film after the L-RB film is immersed in NaTPBF solution containing a buffer solution (pH 4.0) for 30 min. The absorbance of the L-RB film at 559 nm shown in Figure 1 increased as the concentration of NaTPBF increases in the 0 to 10.0 μM region (*r*^2^ = 0.980), as indicated in Figure 2. The graph equation for NaTPBF was 0.0081 *x* + 0.0296. Here, *x* is the µM concentration of NaTPBF, and *y* is the absorbance of the L-RB film. Next, the fluorescence of the L-RB film was measured in order to determine NaTPBF in the lower concentration region. Figure 3 depicts the excitation and fluorescence spectra of the L-RB film after it was immersing to a 5.0 µM NaTPBF solution (pH 4.0, adjusted with a 0.1 M CH_3_COOH/CH_3_COONa buffer solution) and the pH 4.0 buffer solution (0 µM NaTPBF solution) for 30 min, respectively. It can be seen that in case the sensing film is immersed in the NaTPBF solution, the fluorescence intensity of the sensing film significantly increased, compared with that of the case of immersion of the sensing film to pH 4.0 buffer solution. When the L-RB film was immersed in a NaTPBF solution, the maximum fluorescence intensity of the L-RB film was obtained at λ_ex_ = 561 nm and λ_em_ = 584 nm. In the subsequent experiments, the wavelengths were used for the fluorescence intensity measurements of the L-RB film. The increase of the fluorescence intensity at λ_ex_ = 561 nm and λ_em_ = 584 nm for the L-RB film immersed to the 5.0 µM NaTPBF solution was apparent due to a coextraction of the TPBF^−^ anion and a proton into the L-RB film. Here, the L-RB protonates and associates with the extracted TPBF^−^ anion; as a result, the protonation of the L-RB results in a spectral change. The formation reaction of the ion associate between the protonated L-RB and extracted TPBF^−^ anion was as follows:

(TPBF^−^)_aq_ + (H^+^)_aq_ + (L-RB)_memb_ (low fluorescence intensity) ⇄ (L-RB-H^+^-TPBF^−^)_memb_ (high fluorescence intensity)

where memb is the L-RB film, and aq is an aqueous solution.

The fluorescence intensity of the L-RB film also increases as the immersion time of the L-RB film to the NaTPBF solution increases. This may be due to the very low extraction rate of NaTPBF, which is similar to that of anionic surfactants, into the L-RB film and hence, a very long response time is obtained. The drawback of the very long response time of the optode was solved by a kinetic approach in which the fluorescence intensity of the optode is measured at a fixed time. An immersion time of 10 min was used in subsequent fluorescence experiments as a compromise of the sensitivity of the L-RB film and sample throughput. A good linear relationship (*r*^2^ = 0.980) was also obtained between the fluorescence intensity at 561 nm and the NaTPBF concentration in the 0 to 10.0 μM range.

### 2.2. Calibration Curve for PHMB

**Figure 4:**
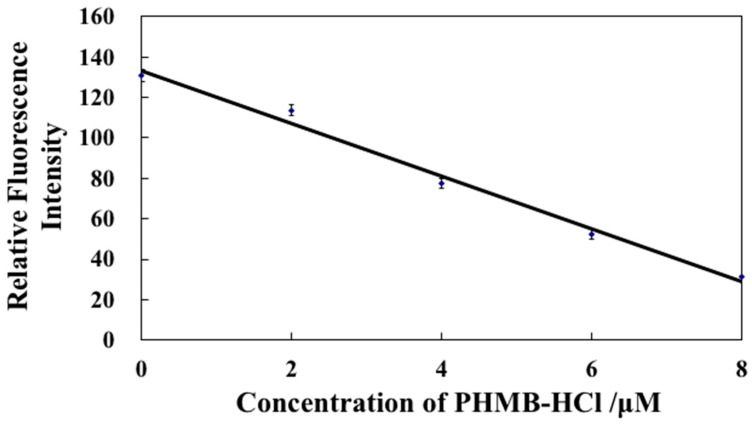
Calibration curve for polyhexamethylene biguanide hydrochloride (PHMB) in the presence of 20.0 μM NaTPBF obtained by using an L-RB-based fluorescence optode.

The effect of a coexisting concentration of NaTPBF in a sample solution on the response sensitivity of the L-RB film to the PHMB anion was examined. If 100.0 μM was used as the coexisting concentration of NaTPBF, the sensitivity of the optode was much less than that when 10.0 and 20.0 μM are used as the coexisting concentrations of NaTPBF. In the event that 10.0 and 20.0 μM are used as coexisting concentrations of NaTPBF, the sensitivity of the optode is almost the same. When 10.0 μM is used as the coexisting concentration of NaTPBF, the concentration of PHMB in the commercially available contact lens detergents was not agreement with its nominal concentration in a preliminary examination (see Section 2.4). Since the determination concentration range of the optode for PHMB is enough to measure PHMB in the commercially available CLDs (*ca.* 1.1 ppm, 5.0 μM) when 20 μM is used for the coexisting concentration of NaTPBF, other concentrations of NaTPBF were not investigated. From the finding, the concentration of NaTPBF was determined to be 20.0 µM. Figure 4 reveals a standard calibration curve for PHMB in three measurements, which were obtained by using the L-RB film measured at λ_ex_ = 561 nm and λ_em_ = 584 nm after the immersion of the L-RB film to a PHMB solution in the presence of 20 μM NaTPBF for 10 min. The error bars show the average ± relative standard deviation. A plot of the fluorescence intensity of the L-RB film versus the concentration of PHMB ranged from 0 to 8.0 µM yields a straight line (*r*^2^ = 0.989). The regression equation of the calibration curve for PHMB is *y* = −13.0 *x* + 133. Here, *x* is the µM concentration of PHMB, and *y* is the relative fluorescence intensity. The detection limit of the optode, which is defined as the concentration equivalent to 3σ, was ca. 1.6 µM, where σ is the standard deviation of the response for a blank solution.

The tetrahydrofuran (THF) solution of the components of the L-RB film was stored for at least six months in a refrigerator at 4 °C while maintaining its performance as a sensing film for PHMB.

### 2.3. Evaluation of the Removal of PHMB by Using Several Solid-Phase Extraction Columns

Commercially available CLDs often contain polyhexanide (PHMB, 1.1 ppm, equivalent to 5.0 μM), buffer agent, stabilizer, an isotonic agent, pH adjuster, poloxamine (nonionic surfactant), and an ingredient called hydranate, which is known by chemists as hydroxyalkylphosphonate. In order to quantitate PHMB in CLDs by utilizing a standard addition method, the CLDs containing no PHMB must be prepared. Therefore, the removal of PHMB using several solid-phase extraction (SPE) columns was evaluated for 6.0 μM PHMB as a sample solution. Table 1 shows the sequence of adsorption of PHMB by solid-phase extraction columns. The sequence is as follows: Sep-Pak Plus CN > Sep-Pak Plus C18 > Sep-Pak Plus C8 > Sep-Pak Plus PS 2 > OASIS HLB 1cc. In case that Sep-Pak Plus CN as a solid-phase extraction column was used, approximately all the PHMB was removed. From the result, Sep-Pak Plus CN as a solid-phase extraction column was used for removal of PHMB in order to quantify PHMB in the CLDs by a standard addition method.

### 2.4. Determination of PHMB in the CLDs

**Figure 5:**
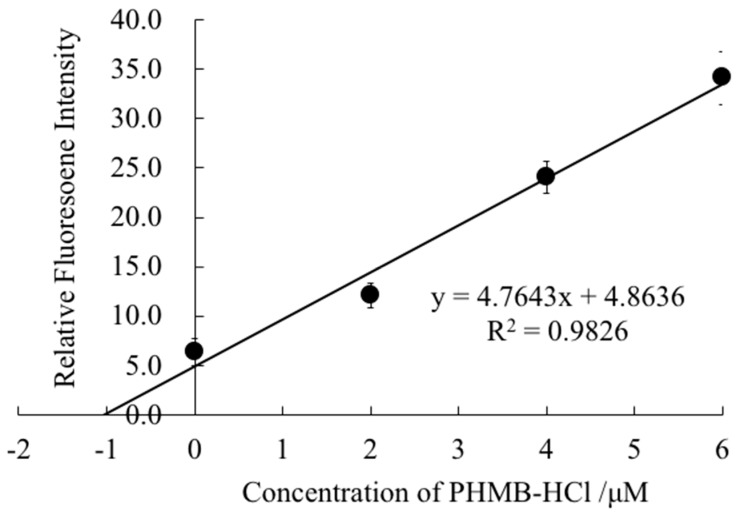
Determination of PHMB in a contact lens detergent (A) by the standard addition method. The fluorescence intensity for a solution containing 20.0 μM of NaTPBF and a fivefold diluted contact lens detergent solution (A) through a Sep-Pak CN column was deducted from that for the solutions containing 20.0 μM of NaTPBF and a known amount of PHMB to a fivefold diluted contact lens detergent solution (A) without a Sep-Pak CN column. The deducted fluorescence intensity as the vertical axis was plotted for added PHMB concentration as the horizontal axis.

The fluorescence intensities of the L-RB film for the solutions containing 20.0 μM of NaTPBF and a known amount of PHMB to a fivefold diluted CLD solution (A), and those for a solution containing 20.0 μM NaTPBF and a fivefold diluted CLD solution (A) through a Sep-Pak CN column were measured, respectively. The fluorescence intensity for a solution containing 20.0 μM NaTPBF and a fivefold diluted CLD solution (A) through a Sep-Pak CN column was deducted from that for the solutions containing 20.0 μM NaTPBF and a known amount of PHMB to a fivefold diluted contact lens detergent solution (A) without a Sep-Pak CN column. Figure 5 shows a plot of the deducted fluorescence intensity as the vertical axis with added PHMB concentration as the horizontal axis. From the X-intercept of the straight line, the concentration of PHMB in the CLD (A) is calculated to be 5.1 μM, which is similar to the nominal PHMB concentration (5.0 μM). Table 2 shows results for the determination of PHMB in CLD (A) and (B). From Table 1, the L-RB film-based optode was capable of determining PHMB in a few CLDs. 

## 3. Experimental

### 3.1. Reagents

Poly (vinyl chloride) (PVC) powder and rhodamine B (RB) from Wako Pure Chemical Industries, Ltd. (Osaka, Japan) were used. 2-nitrophenyl octyl ether (NPOE), tetrakis (4-fluorophenyl) borate, sodium salt, and dihydrate (NaTPBF) were purchased from Dojindo Laboratories (Kumamoto, Japan). Sep-Pak Plus CN, Sep-Pak Plus C18, Sep-Pak Plus C8, OASIS HLB 1cc, and Sep-Pak Plus PS 2 purchased from Waters (Milford, MA, United States) were used as the solid-phase extraction (SPE) columns for the removal of PHMB. All other chemicals of reagent grade were used as received. The PHMB concentration (M = mol dm^−3^) indicates the number of moles of ionic groups/volume (liter) of polymer solution.

### 3.2. Preparation of Optode Film Containing L-RB

An optode film containing L-RB was prepared as follows. The fabrication method is similar to our previous paper [14,15,16]. Take NPOE (5.0 g) and 24.0 mL of a mixed aqueous solution containing 75.0 μM RB and 1.0 M NaOH in a plastic tube, stir for 24 h at 55 °C, and extract L-RB into the NPOE phase. The NPOE phase (4.0 g) centrifuged from the aqueous phase (10 min) and PVC powder (0.80 g) were solubilized in 20 mL of tetrahydrofuran (THF). By means of immersing a quartz glass plate (length: 3.5 cm, width: 1.0 cm, thickness: 1 mm) in the THF solution for 5 s, an L-RB film for the measurement of fluorescence was fabricated. In the event of measurement of absorbance of the L-RB film, a glass plate (length of 3.5 cm, a width of 1.0 cm and thickness of 1 mm) was used in place of the quartz glass plate. The L-RB film was dried at room temperature for more than 2 h and then conditioned with CH_3_COOH-CH_3_COONa buffer solution (pH 4.0) for 24 h before starting an experiment. The fluorescence intensity of the L-RB film was measured for 10 min at λ_ex_ = 561 nm and λ_em_ = 584 nm on a spectrofluorimeter (JASCO FP-750, JASCO Corporation, Tokyo, Japan). The measurement was performed by placing the L-RB film on the diagonal of a 1 cm square quartz cuvette (height 4.5 cm, length 1.25 cm, width 1.25 cm) containing a sample solution (3.0 mL). The thickness of the prepared L-RB film was about 0.2 mm. In the case of measurement of absorbance of the L-RB film, glass plates coated with L-RB films were fixed in disposable cuvettes (4.5 cm high, 1.25 cm long, and 1.25 cm wide) filled with 2.0 mL of PHMB solution (pH 4.0 adjusted with a 0.1M CH_3_COOH/CH_3_COONa buffer solution). The absorption spectrum of the L-RB films was measured with a spectrophotometer (JASCO, V-530-iRM, JASCO Corporation, Tokyo, Japan). All spectroscopic measurements were performed in batch mode. The L-RB film was used as a disposable sensor for one-shot measurement.

### 3.3. Evaluation of Removal of PHMB by Using Several Solid-Phase Extraction Columns with a Flow Injection Analysis (FIA)

The FIA system and its experimental conditions for evaluation of the removal of PHMB in commercially available CLDs using several solid-phase extraction (SPE) columns are the same as that for the measurement of PHMB in the previous paper [11]. A PHMB sample solution (140 μL) with and without pretreatment using the SPE columns was injected into the FIA system. The peak height for 6.0 μM PHMB with the SPE columns was compared with that without those columns, and the concentration of adsorbed PHMB in the columns was measured from the calibration curve for PHMB without the columns.

## 4. Conclusions

A fluorescence optode based on an L-RB film for the determination of PHMB was fabricated. The response of the optode shows a good linear response between fluorescence intensity and the concentration of PHMB with the concentration range of 0 to 8.0 μM. The application of the L-RB film optode was made for the determination of PHMB in the CLDs. The procedures of the proposed method based on the L-RB film optode is much simpler than those of the conventional determination methods without using very toxic reagents. The L-RB film is easily fabricated with relatively low cost. The present method will be very promising for the quality control of CLDs. The L-RB film was used as a reversible film for the FIA of AS [16]. Therefore, the L-RB film will be used as a reversible film for the determination of PHMB. We continue the research regarding the FIA of PHMB using the optode detector based on L-RB film as a reversible film.

## Figures and Tables

**Table 1:** Evaluation of the removal of PHMB by using several solid-phase extraction columns.

Solid-Phase Extraction Column	Concentration of Adsorbed PHMB Column	Removal of PHMB in from the Column/%
Sep-Pak Plus CN	6.0 μM	100
Sep-Pak Plus C18	5.0 μM	83
Sep-Pak Plus C8	2.8 μM	47
OASIS HLB 1cc	1.0 μM	17
Sep-Pak Plus PS 2	2.1 μM	35

The peak height for 6.0 μM PHMB with the SPE columns was compared with that without those columns, and the concentration of adsorbed PHMB in the columns was measured from the calibration curve for PHMB without the columns.

**Table 2:** Determination of PHMB in contact lens detergents.

Contact Lens Detergents	Nominal Value	Determination Value/μM
(A)	1.1 ppm (5.0 µM)	5.1
(B)	0.001 mg/mL (4.6 µM)	4.8
